# Global Transcriptional Response to *Hfe* Deficiency and Dietary Iron Overload in Mouse Liver and Duodenum

**DOI:** 10.1371/journal.pone.0007212

**Published:** 2009-09-29

**Authors:** Alejandra Rodriguez, Tiina Luukkaala, Robert E. Fleming, Robert S. Britton, Bruce R. Bacon, Seppo Parkkila

**Affiliations:** 1 Institute of Medical Technology, University of Tampere and Tampere University Hospital, Tampere, Finland; 2 Science Center, Pirkanmaa Hospital District and Tampere School of Public Health, University of Tampere, Tampere, Finland; 3 Department of Pediatrics, Saint Louis University School of Medicine, St. Louis, Missouri, United States of America; 4 Edward A. Doisy Department of Biochemistry and Molecular Biology, Saint Louis University School of Medicine, St. Louis, Missouri, United States of America; 5 Saint Louis University Liver Center, St. Louis, Missouri, United States of America; 6 Division of Gastroenterology and Hepatology, Department of Internal Medicine, Saint Louis University School of Medicine, St. Louis, Missouri, United States of America; 7 School of Medicine, University of Tampere and Tampere University Hospital, Tampere, Finland; Centre de Regulació Genòmica, Spain

## Abstract

Iron is an essential trace element whose absorption is usually tightly regulated in the duodenum. *HFE*-related hereditary hemochromatosis (HH) is characterized by abnormally low expression of the iron-regulatory hormone, hepcidin, which results in increased iron absorption. The liver is crucial for iron homeostasis as it is the main production site of hepcidin. The aim of this study was to explore and compare the genome-wide transcriptome response to *Hfe* deficiency and dietary iron overload in murine liver and duodenum. Illumina™ arrays containing over 47,000 probes were used to study global transcriptional changes. Quantitative RT-PCR (Q-RT-PCR) was used to validate the microarray results. In the liver, the expression of 151 genes was altered in *Hfe^−/−^* mice while dietary iron overload changed the expression of 218 genes. There were 173 and 108 differentially expressed genes in the duodenum of *Hfe^−/−^* mice and mice with dietary iron overload, respectively. There was 93.5% concordance between the results obtained by microarray analysis and Q-RT-PCR. Overexpression of genes for acute phase reactants in the liver and a strong induction of digestive enzyme genes in the duodenum were characteristic of the *Hfe*-deficient genotype. In contrast, dietary iron overload caused a more pronounced change of gene expression responsive to oxidative stress. In conclusion, *Hfe* deficiency caused a previously unrecognized increase in gene expression of hepatic acute phase proteins and duodenal digestive enzymes.

## Introduction

Iron plays crucial roles in cellular metabolism but, in excess, it can catalyze the formation of free radicals leading to oxidative stress and cell damage [1]. Iron is absorbed in the duodenum, where it crosses the apical and basolateral membranes of absorptive enterocytes to enter the blood stream [2]. There is no regulated mechanism of iron excretion, and thus the absorption of iron must be tightly regulated to maintain iron balance. *HFE*-related hereditary hemochromatosis (HH, OMIM-235200) is an autosomal recessive disorder in which absorption of iron is inappropriately high [3], [4]. HH is characterized by high transferrin saturation and low iron content in macrophages. Iron is deposited primarily in the parenchymal cells of various organs, particularly the liver, but also the pancreas, heart, skin, and testes, resulting in tissue damage and organ failure. Clinical complications in untreated HH patients include hepatic fibrosis, cirrhosis, hepatocellular carcinoma, diabetes, cardiomyopathy, hypogonadism, and arthritis [4].

HH is characterized by inappropriately low expression of the iron-regulatory hormone hepcidin [5]. Hepcidin, a small peptide hormone expressed mainly in the liver, is a central player in the maintenance of iron balance [6]. The only known molecule capable of transporting iron out of cells is ferroportin [7]–[9]. This iron exporter is located in the plasma membrane of enterocytes, reticuloendothelial cells, hepatocytes, and placental cells [7]. Hepcidin binds to ferroportin and induces its internalization and degradation, therefore suppressing the transport of iron into the circulation [10]. The expression of hepcidin is induced by increased iron stores and inflammation, and suppressed by hypoxia and anemia [11], [12].

Mice homozygous for a null allele of *Hfe* (*Hfe^−/−^*) provide a genetic animal model of HH [13]. There are several animal models of iron overload based on administration of exogenous iron [14]. According to the route of iron delivery, these can be divided into two main types: enteral (i.e. dietary) and parenteral models. For example, dietary supplementation with carbonyl iron in mice reproduces the HH pattern of hepatic iron loading, with predominantly parenchymal iron deposition [14]. Although both *Hfe* ^−/−^ mice and carbonyl iron-fed mice develop iron overload, there are important differences between these two models. *Hfe* ^−/−^ mice lack Hfe protein and therefore have decreased expression of hepcidin [15], [16], while mice with dietary iron overload express functional Hfe protein and their hepcidin expression is elevated [12].

Current RNA microarray technology allows expression profiling of the whole transcriptome. This methodology has been used to explore the effects of *Hfe* gene disruption on mRNA expression in the liver and duodenum, two organs with crucial roles in iron metabolism [17]. In the present study, we used this approach to study gene expression in the liver and duodenum of *Hfe* ^−/−^ mice and wild-type mice, with or without dietary iron overload. This allowed the identification of genes whose expression is changed during iron overload and those genes whose expression is differentially influenced by lack of Hfe protein.

## Results

We used global microarray analysis to study gene expression in the liver and duodenum of *Hfe* ^−/−^ mice and carbonyl iron loaded mice, and comparing it with that of wild-type mice fed a standard diet. This approach allowed the identification of genes whose expression is changed during iron overload and those genes whose expression is differentially influenced by lack of Hfe protein. All the mice used were males and all had the same genetic background (C57BL/6).

### Hepatic transcriptional response to *Hfe* deficiency and dietary iron overload

Hepatic RNA from 3 *Hfe^−/−^* mice and 2 wild-type mice was subjected to microarray analysis. The Pearson correlation coefficient between the knock out mice and between the controls was in both cases 0.989. The results revealed 86 induced genes and 65 repressed genes, using a cutoff value of ±1.4-fold (Table 1 and Dataset S1). This cutoff value has been proposed as an adequate compromise above which there is a high correlation between microarray and Q-RT-PCR data, regardless of other factors such as spot intensity and cycle threshold [18]. The fold-changes ranged from 9.83 to −3.47. Functional annotation of the gene lists highlighted the biological processes that may be modified by *Hfe* deficiency. This analysis revealed enrichment of heat shock proteins and proteins related to inflammatory responses or antigen processing and presentation, among others (Table 2).

**Table 1:** Number of genes regulated by *Hfe* deficiency or dietary iron overload in murine liver and duodenum.

Tissue	Model	Total regulated genes	Upregulated genes	Downregulated genes	Proportion of results confirmed by Q-RT-PCR
**Liver**	*Hfe* ^−/−^	151	86	65	11/12
	Dietary Iron	218	123	95	16/17
**Duodenum**	*Hfe* ^−/−^	173	143	30	6/7
	Dietary Iron	108	49	59	10/10

Genes with changes in mRNA expression greater than ±1.4-fold were considered as regulated.

**Table 2:** Functional annotation of genes regulated in the liver of *Hfe^−/−^* mice.

Functional Category	Gene Symbol	Description	GenBank Number	FC	Q-PCR
Response to unfolded protein	Hspd1	heat shock protein 1 (chaperonin)	NM_010477	1.54	
	H47	histocompatibility 47	NM_024439	−1.45	
	Hsp90ab1	heat shock protein 90 kDa alpha (cytosolic), class B member 1	NM_008302	−1.48	
	Hspb1	heat shock protein 1	NM_013560	−1.66	
	Hspa8	heat shock protein 8	NM_031165	−1.70	
	Hsp90b1	heat shock protein 90 kDa beta (Grp94), member 1	NM_011631	−1.71	
	Hsp90aa1	heat shock protein 90 kDa alpha (cytosolic), class A member 1	NM_010480	−1.72	
	Hspa5	heat shock protein 5	NM_022310	−2.14	
	Hsph1	heat shock 105 kDa/110 kDa protein 1	NM_013559	−2.16	−2.43
	Syvn1	synovial apoptosis inhibitor 1, synoviolin	NM_028769	−2.45	
Inflammatory response	Saa2	serum amyloid A 2	NM_011314	9.83	39.36
	Saa1	serum amyloid A 1	NM_009117	6.30	16.36
	Orm2	orosomucoid 2	NM_011016	3.29	
	Saa3	serum amyloid A 3	NM_011315	2.89	
	Orm1	orosomucoid 1	NM_008768	1.68	
	Serpina3n	serine (or cysteine) peptidase inhibitor, clade A, member 3N	NM_009252	1.63	
	C1s	complement component 1, s subcomponent	NM_144938	1.47	
	Cxcl9	chemokine (C-X-C motif) ligand 9	NM_008599	−1.57	
Apolipoprotein associated with HDL	Saa2	serum amyloid A 2	NM_011314	9.83	39.36
	Saa1	serum amyloid A 1	NM_009117	6.30	16.36
	Saa3	serum amyloid A 3	NM_011315	2.89	
	Apoa4	apolipoprotein A-IV	NM_007468	2.36	
Monooxygenase activity	Moxd1	monooxygenase, DBH-like 1	NM_021509	4.12	
	Cyp2a5	cytochrome P450, family 2, subfamily a, polypeptide 5	NM_007812	1.67	
	Cyp27a1	cytochrome P450, family 27, subfamily a, polypeptide 1	NM_024264	1.64	
	Cyp2d26	cytochrome P450, family 2, subfamily d, polypeptide 26	NM_029562	1.59	
	Kmo	kynurenine 3-monooxygenase (kynurenine 3-hydroxylase)	NM_133809	1.48	
	Cyp4a14	cytochrome P450, family 4, subfamily a, polypeptide 14	NM_007822	−1.44	
	Cyp3a11	cytochrome P450, family 3, subfamily a, polypeptide 11	NM_007818	−1.58	
	Cyp26b1	cytochrome P450, family 26, subfamily b, polypeptide 1	NM_175475	−2.39	−2.18
Steroid biosynthetic process	Nsdhl	NAD(P) dependent steroid dehydrogenase-like	NM_010941	1.44	
	Hmgcs1	3-hydroxy-3-methylglutaryl-Coenzyme A synthase 1	NM_145942	−1.42	
	Lss	lanosterol synthase	NM_146006	−1.45	
	Hmgcr	3-hydroxy-3-methylglutaryl-Coenzyme A reductase	NM_008255	−1.50	
	Mvd	mevalonate (diphospho) decarboxylase	NM_138656	−1.67	
Antigen processing and presentation	Psmb8	proteasome (prosome, macropain) subunit, beta type 8 (large multifunctional peptidase 7)	NM_010724	1.50	
	Cd74	CD74 antigen (invariant polypeptide of major histocompatibility complex, class II antigen-associated)	NM_010545	−1.59	
	H2-Eb1	histocompatibility 2, class II antigen E beta	NM_010382	−1.63	
	H2-Ab1	histocompatibility 2, class II antigen A, beta 1	NM_207105	−1.77	
	H2-Aa	histocompatibility 2, class II antigen A, alpha	NM_010378	−1.81	
Endopeptidase inhibitor activity	Serpina12	serine (or cysteine) peptidase inhibitor, clade A (alpha-1 antiproteinase, antitrypsin), member 12	NM_026535	2.01	
	Wfdc2	WAP four-disulfide core domain 2	NM_026323	1.65	
	Serpina3n	serine (or cysteine) peptidase inhibitor, clade A, member 3N	NM_009252	1.63	
	Itih4	inter alpha-trypsin inhibitor, heavy chain 4	NM_018746	1.48	
carboxy-lyase activity	Ddc	dopa decarboxylase	NM_016672	−1.48	
	Mvd	mevalonate (diphospho) decarboxylase	NM_138656	−1.67	
	Csad	cysteine sulfinic acid decarboxylase	NM_144942	−1.76	
T cell differentiation	Cd74	CD74 antigen (invariant polypeptide of major histocompatibility complex, class II antigen-associated)	NM_010545	−1.59	
	Hsp90aa1	heat shock protein 90 kDa alpha (cytosolic), class A member 1	NM_010480	−1.72	
	Egr1	early growth response 1	NM_007913	−1.77	
	H2-Aa	histocompatibility 2, class II antigen A, alpha	NM_010378	−1.81	
	Gadd45g	growth arrest and DNA-damage-inducible 45 gamma	NM_011817	−1.97	
Glycogen metabolic process	G6pc	glucose-6-phosphatase, catalytic	NM_008061	2.38	
	Ppp1r3c	protein phosphatase 1, regulatory (inhibitor) subunit 3C	NM_016854	1.57	
	Ppp1r3b	protein phosphatase 1, regulatory (inhibitor) subunit 3B	NM_177741	1.55	

Another microarray experiment was performed using hepatic RNA from 3 mice with dietary iron overload and 2 mice fed a standard diet. The similarity between samples from individual mice was measured as the Pearson correlation coefficient, which was 0.989 between iron overloaded mice and 0.991 between control mice. The expression of 123 genes was upregulated and that of 95 genes was downregulated, applying a cutoff value of ±1.4-fold (Table 1 and Dataset S2). The fold-changes ranged between 13.58 and −7.46. The list of regulated genes was functionally annotated (Table 3), showing enrichment of cytochrome P450 proteins as well as others involved in glutathione metabolism, acute-phase response, organic acid biosynthetic process and cellular iron homeostasis, among others.

**Table 3:** Functional annotation of genes regulated in the liver of iron-fed mice.

Functional Category	Gene Symbol	Description	GenBank Number	FC	Q-PCR
Electron transport, containing heme and monooxygenase activity	Cyp2b10	cytochrome P450, family 2, subfamily b, polypeptide 10	NM_009999	13.58	
	Cyp2b9	cytochrome P450, family 2, subfamily b, polypeptide 9	NM_010000	7.41	
	Cyp4a14	cytochrome P450, family 4, subfamily a, polypeptide 14	NM_007822	6.97	16.06
	Cyp26b1	cytochrome P450, family 26, subfamily b, polypeptide 1	NM_175475	2.24	
	Cyp2c29	cytochrome P450, family 2, subfamily c, polypeptide 29	NM_007815	1.77	
	Cyp2c54	cytochrome P450, family 2, subfamily c, polypeptide 54	NM_206537	1.76	2.37
	Cyp2a5	cytochrome P450, family 2, subfamily a, polypeptide 5	NM_007812	1.65	
	Cyp2b13	cytochrome P450, family 2, subfamily b, polypeptide 13	NM_007813	1.50	
	Cyp4v3	cytochrome P450, family 4, subfamily v, polypeptide 3	NM_133969	−1.82	
	Cyp7b1	cytochrome P450, family 7, subfamily b, polypeptide 1	NM_007825	−2.50	
	Cyp4a12b	cytochrome P450, family 4, subfamily a, polypeptide 12B	NM_172306	−2.73	
	Cyp7a1	cytochrome P450, family 7, subfamily a, polypeptide 1	NM_007824	−2.80	
	Cyp4a12a	cytochrome P450, family 4, subfamily a, polypeptide 12a	NM_177406	−3.62	
Glutathione metabolism	Gsta1	glutathione S-transferase, alpha 1 (Ya)	NM_008181	1.94	
	Gstt2	glutathione S-transferase, theta 2	AK079739	1.86	
	Gsta2	glutathione S-transferase, alpha 2 (Yc2)	NM_008182	1.83	
	Gstm6	glutathione S-transferase, mu 6	NM_008184	1.78	
	Mgst3	microsomal glutathione S-transferase 3	NM_025569	1.72	
	Gstm3	glutathione S-transferase, mu 3	NM_010359	1.59	
	Gclc	glutamate-cysteine ligase, catalytic subunit	NM_010295	1.55	
	Gstp1	glutathione S-transferase, pi 1	NM_013541	−1.81	
Acute-phase response	Il1b	interleukin 1 beta	NM_008361	2.04	
	Saa3	serum amyloid A 3	NM_011315	−1.82	
	Saa4	serum amyloid A 4	NM_011316	−1.91	
	Saa2	serum amyloid A 2	NM_011314	−2.79	−3.36
	Saa1	serum amyloid A 1	NM_009117	−3.96	−4.31
Organic acid biosynthetic process	Fasn	fatty acid synthase	NM_007988	2.22	
	Elovl6	ELOVL family member 6, elongation of long chain fatty acids	NM_130450	1.87	
	Acaca	acetyl-Coenzyme A carboxylase alpha	NM_133360	1.81	
	Cd74	CD74 antigen (invariant polypeptide of major histocompatibility complex, class II antigen-associated)	NM_010545	1.65	
	Cyp7b1	cytochrome P450, family 7, subfamily b, polypeptide 1	NM_007825	−2.50	
	Elovl3	elongation of very long chain fatty acids-like 3	NM_007703	−5.00	
Cellular iron ion homeostasis	Hamp2	hepcidin antimicrobial peptide 2	NM_183257	10.03	24.77
	Hamp1	hepcidin antimicrobial peptide 1	NM_032541	1.73	5.27
	Tfrc	transferrin receptor	NM_011638	−1.74	
	Alas2	aminolevulinic acid synthase 2, erythroid	NM_009653	−2.20	
Hemopoiesis and immune system development	Id2	inhibitor of DNA binding 2	NM_010496	2.92	5.2
	Egr1	early growth response 1	NM_007913	2.55	
	H2-Aa	histocompatibility 2, class II antigen A, alpha	NM_010378	1.81	
	Gadd45g	growth arrest and DNA-damage-inducible 45 gamma	NM_011817	1.66	
	Cd74	CD74 antigen (invariant polypeptide of major histocompatibility complex, class II antigen-associated)	NM_010545	1.65	
	Hbb-b1	hemoglobin, beta adult major chain	NM_008220	1.45	
	Pik3r1	phosphatidylinositol 3-kinase, regulatory subunit, polypeptide 1 (p85 alpha), transcript variant 1	NM_001024955	−1.70	
	Alas2	aminolevulinic acid synthase 2, erythroid	NM_009653	−2.20	
	Bcl6	B-cell leukemia/lymphoma 6	NM_009744	−2.61	
Serine-type endopeptidase inhibitor activity	Serpina7	serine (or cysteine) peptidase inhibitor, clade A (alpha-1 antiproteinase, antitrypsin), member 7	NM_177920	2.12	
	Serpina3m	serine (or cysteine) peptidase inhibitor, clade A, member 3 M	NM_009253	2.04	
	Spink4	serine peptidase inhibitor, Kazal type 4	NM_011463	1.52	
	Serpina1e	serine (or cysteine) peptidase inhibitor, clade A, member 1e	NM_009247	−1.86	
	Serpina12	serine (or cysteine) peptidase inhibitor, clade A (alpha-1 antiproteinase, antitrypsin), member 12	NM_026535	−2.19	
	Serpine2	serine (or cysteine) peptidase inhibitor, clade E, member 2	AK045954	−2.88	
Antigen processing and presentation via MHC class II	H2-Aa	histocompatibility 2, class II antigen A, alpha	NM_010378	1.81	
	H2-Ab1	histocompatibility 2, class II antigen A, beta 1	NM_207105	1.68	
	Cd74	CD74 antigen (invariant polypeptide of major histocompatibility complex, class II antigen-associated)	NM_010545	1.65	
	H2-Eb1	histocompatibility 2, class II antigen E beta	NM_010382	1.43	

There were 11 upregulated and 7 downregulated genes that were affected by both *Hfe* deficiency and dietary iron overload in similar fashion, while 27 genes were regulated in opposite directions by these two conditions in the liver (Table 4). In some cases, several genes belonging to the same gene family showed divergent regulation (e.g., *Saa1*, *Saa2*, *Saa3*) with upregulation in *Hfe^−/−^* mice and downregulation by dietary iron overload.

**Table 4:** Comparison of hepatic gene regulation by *Hfe* deficiency or dietary iron overload.

	Gene Symbol	Description	GenBank Number	FC Hfe^−/−^	FC diet
Increased in Hfe^−/−^ and by diet	*Lcn2*	lipocalin 2	NM_008491	9.54	2.10
	*Rgs16*	regulator of G-protein signaling 16	NM_011267	4.61	5.06
	*Mt1*	metallothionein 1	NM_013602	4.17	3.95
	*Apoa4*	apolipoprotein A-IV	NM_007468	2.36	6.56
	*Slc2a2*	solute carrier family 2 (facilitated glucose transporter), member 2	NM_031197	1.92	2.17
	*Mfsd2*	major facilitator superfamily domain containing 2	NM_029662	1.68	3.59
	*Cyp2a5*	cytochrome P450, family 2, subfamily a, polypeptide 5	NM_007812	1.67	1.65
	*Gstt2*	glutathione S-transferase, theta 2	NM_010361	1.58	1.86
	*Ppp1r3c*	protein phosphatase 1, regulatory (inhibitor) subunit 3C	NM_016854	1.57	1.53
	*Bhlhb2*	basic helix-loop-helix domain containing, class B2	NM_011498	1.52	2.35
	*Dusp1*	dual specificity phosphatase 1	NM_013642	1.50	2.15
Increased in Hfe^−/−^ and decreased by diet	*Saa2*	serum amyloid A 2	NM_011314	9.83	−2.79
	*Saa1*	serum amyloid A 1	NM_009117	6.30	−3.96
	*Saa3*	serum amyloid A 3	NM_011315	2.89	−1.82
	*Angptl4*	angiopoietin-like 4	NM_020581	2.30	−2.03
	*Hp*	haptoglobin	NM_017370	2.23	−1.69
	*Serpina12*	serine (or cysteine) peptidase inhibitor, clade A (alpha-1 antiproteinase, antitrypsin), member 12	NM_026535	2.01	−2.19
	*Lpin1*	lipin 1	NM_015763	1.92	−1.59
	*Il6ra*	interleukin 6 receptor, alpha	AK020663	1.70	−2.08
	*Dio1*	deiodinase, iodothyronine, type I	NM_007860	1.57	−1.87
	*Ppp1r3b*	protein phosphatase 1, regulatory (inhibitor) subunit 3B	NM_177741	1.55	−1.95
	*Dct*	dopachrome tautomerase	NM_010024	1.50	−2.72
	*Mup4*	major urinary protein 4	NM_008648	1.44	−4.28
Decreased in Hfe^−/−^ and increased by diet	*Cyp26b1*	cytochrome P450, family 26, subfamily b, polypeptide 1	NM_175475	−2.39	2.24
	*Phlda1*	pleckstrin homology-like domain, family A, member 1	NM_009344	−2.20	1.51
	*Gadd45g*	growth arrest and DNA-damage-inducible 45 gamma	NM_011817	−1.97	1.66
	*Socs3*	suppressor of cytokine signaling 3	NM_007707	−1.96	1.89
	*Cish*	cytokine inducible SH2-containing protein	NM_009895	−1.93	2.37
	*H2-Aa*	histocompatibility 2, class II antigen A, alpha	NM_010378	−1.81	1.81
	*Egr1*	early growth response 1	NM_007913	−1.77	2.55
	*H2-Ab1*	histocompatibility 2, class II antigen A, beta 1	NM_207105	−1.77	1.68
	*Gsta2*	glutathione S-transferase, alpha 2 (Yc2)	NM_008182	−1.71	1.83
	*H2-Eb1*	histocompatibility 2, class II antigen E beta	NM_010382	−1.63	1.43
	*Cd74*	CD74 antigen (invariant polypeptide of major histocompatibility complex, class II antigen-associated)	NM_010545	−1.59	1.65
	*Cyp4a14*	cytochrome P450, family 4, subfamily a, polypeptide 14	NM_007822	−1.44	6.97
	*Hbb-b1*	hemoglobin, beta adult major chain	AK010993	−1.42	1.45
	*Rnf186*	ring finger protein 186	NM_025786	−1.41	1.81
	*Hamp1*	hepcidin antimicrobial peptide 1	NM_032541	−1.41	1.73
Decreased in Hfe^−/−^ and by diet	*Creld2*	cysteine-rich with EGF-like domains 2	NM_029720	−3.47	−1.64
	*Hsph1*	heat shock 105 kDa/110 kDa protein 1	NM_013559	−2.16	−2.13
	*Tfrc*	transferrin receptor	NM_011638	−1.92	−1.74
	*Hspb1*	heat shock protein 1	NM_013560	−1.66	−1.81
	*Hhex*	hematopoietically expressed homeobox	NM_008245	−1.55	−2.05
	*Mcm10*	minichromosome maintenance deficient 10 (S. cerevisiae)	NM_027290	−1.55	−1.55
	*Ddc*	dopa decarboxylase	NM_016672	−1.48	−1.97

FC, fold-change; diet, iron-supplemented diet.

#### Altered expression of iron-related genes in the liver

**Figure 1:**
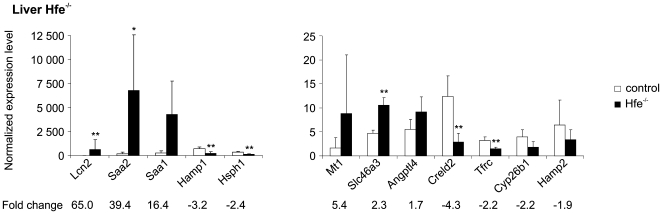
Validation of liver microarray data from *Hfe^−/−^* mice by Q-RT-PCR. The expression of various mRNA species in 5 *Hfe^−/−^* mice is compared to those in 4 wild-type controls. Each sample was run in triplicate. (mean±SD). *p<0.05; **p<0.025; ***p<0.01.

**Figure 2:**
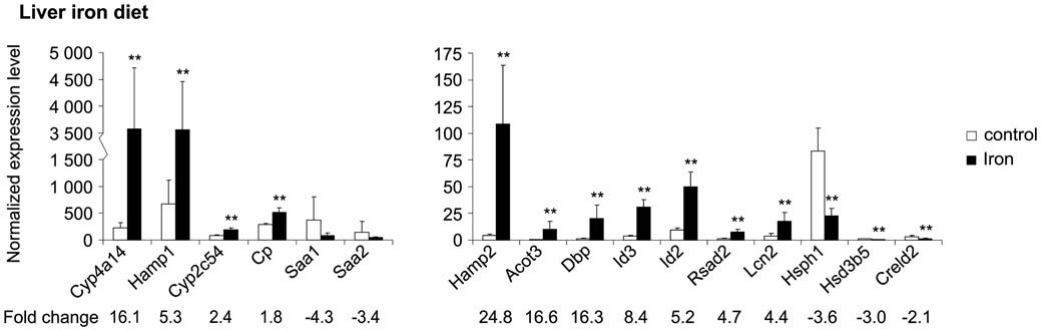
Expression of genes affected by dietary iron overload in the liver, as confirmed by Q-RT-PCR. Samples from 5 mice fed an iron-supplemented diet and 4 mice fed a control diet were used, and each sample was run in triplicate. (mean±SD). *p<0.05; **p<0.025; ***p<0.01.

The expression of 3 iron-related genes was altered in the liver of *Hfe^−/−^* mice. The expression of *Hamp1* and *Tfrc* was decreased and that of *Lcn2* was induced. We confirmed these results using Q-RT-PCR, and also tested the expression of *Hamp2*, which was downregulated (Figure 1). Dietary iron overload changed the expression of 5 iron-related genes in the liver. The expression of *Hamp1*, *Hamp2*, *Lcn2* and *Cp* were upregulated using both microarray analysis and Q-RT-PCR, while *Tfrc* expression was down-regulated by 1.7-fold (Figure 2).

#### Confirmation of hepatic microarray results by Q-RT-PCR

Microarray analysis for the expression of several genes was confirmed by performing Q-RT-PCR on hepatic samples from 5 *Hfe^−/−^* mice, 4 wild-type control mice, 5 iron-fed mice and 4 mice fed a standard diet. For this purpose, we selected iron-related genes and others whose expression was substantially altered in the experimental groups. A total of 29 results from the hepatic microarray data, corresponding to 24 different genes, were tested by Q-RT-PCR, and 27 (93.1%) of them showed concordant results by these two methods (Figures 1 and 2). Changes in *Foxq1* and *Dmt1* expression were false-positives in the microarray analysis for *Hfe^−/−^* mice and dietary iron overload, respectively. The upregulation of *Ltf* expression by dietary iron overload observed by microarray analysis could not be confirmed by Q-RT-PCR because the expression levels in samples from all but one of the treated mice and all control mice were below the detection threshold.

### Duodenal gene expression response to *Hfe* deficiency and dietary iron supplementation

Microarray analysis of duodenal RNA from 2 *Hfe^−/−^* mice and 2 wild-type mice revealed that the expression of 143 genes was upregulated and that of 30 genes was downregulated when a cutoff value of ±1.4-fold was used (Table 1 and Dataset S3). The fold-changes ranged from 15.67 to −3.14. The Pearson correlation coefficient between knockout mice and between controls was 0.976 and 0.971, respectively. Functional categories overrepresented among the genes regulated by *Hfe* deficiency included proteins with endopeptidase activity, and others involved in lipid catabolism and antimicrobial activity (Table 5).

**Table 5:** Functional annotation of genes regulated in the duodenum of *Hfe^−/−^* mice.

Functional Category	Gene Symbol	Description	GenBank Number	FC	Q-PCR
Endopeptidase activity	Ela3	elastase 3, pancreatic	NM_026419	15.67	14.77
	Try4	trypsin 4	NM_011646	13.09	
	RP23-395H4.4	elastase 2A	NM_007919	10.20	
	Ctrl	chymotrypsin-like	NM_023182	9.99	
	Ctrb1	chymotrypsinogen B1	NM_025583	9.68	
	Prss2	protease, serine, 2	NM_009430	7.41	
	2210010C04Rik	RIKEN cDNA 2210010C04 gene	NM_023333	7.14	
	Ela1	elastase 1, pancreatic	NM_033612	5.84	
	Klk1b5	kallikrein 1-related peptidase b5	NM_008456	2.90	
	Ctrc	chymotrypsin C (caldecrin)	NM_001033875	2.51	
	Klk1b11	kallikrein 1-related peptidase b11	NM_010640	2.34	
	Klk1	kallikrein 1	NM_010639	2.29	
	Klk1b27	kallikrein 1-related peptidase b27	NM_020268	2.22	
	Klk1b4	kallikrein 1-related pepidase b4	NM_010915	2.11	
	Klk1b24	kallikrein 1-related peptidase b24	NM_010643	2.10	
	Mela	melanoma antigen	NM_008581	2.05	
	Ctse	cathepsin E	NM_007799	1.91	2.32
	Klk1b26	kallikrein 1-related petidase b26	NM_010644	1.74	
	Capn5	calpain 5	NM_007602	1.60	
Lipid catabolic function	Cel	carboxyl ester lipase	NM_009885	9.82	
	Pnliprp1	pancreatic lipase related protein 1	NM_018874	8.17	
	Clps	colipase, pancreatic	NM_025469	5.35	
	Pla2g1b	phospholipase A2, group IB, pancreas	NM_011107	4.86	
	Pnliprp2	pancreatic lipase-related protein 2	NM_011128	4.50	
	Apoc3	apolipoprotein C-III	NM_023114	−1.79	
Triacylglycerol lipase activity	Cel	carboxyl ester lipase	NM_009885	9.82	
	Pnliprp1	pancreatic lipase related protein 1	NM_018874	8.17	
	Pnliprp2	pancreatic lipase-related protein 2	NM_011128	4.50	
Antimicrobial	Hamp2	hepcidin antimicrobial peptide 2	NM_183257	2.74	6.66
	Defcr-rs1	defensin related sequence cryptdin peptide (paneth cells)	NM_007844	−1.60	
	Lyz1	lysozyme 1	NM_013590	−1.68	
	Defcr6	defensin related cryptdin 6	NM_007852	−2.11	
	Defcr20	defensin related cryptdin 20	NM_183268	−2.69	
Metallocarboxypeptidase activity	Cpa1	carboxypeptidase A1	NM_025350	12.42	
	Cpa2	carboxypeptidase A2, pancreatic	NM_001024698	8.14	
	Cpb1	carboxypeptidase B1 (tissue)	NM_029706	12.51	14.55

Global transcriptional regulation was also studied in the duodenum of mice fed an iron-supplemented diet, using 3 treated mice and 2 controls. The Pearson correlation coefficient was 0.985 between treated mice and 0.983 between controls. The expression of 49 genes was induced and 59 genes were repressed, applying a cutoff value of ±1.4-fold (Table 1 and Dataset S4). The fold-changes ranged between 6.07 and −5.64. Functional annotation of the gene list evidenced enrichment of genes involved in glutathione metabolism, antigen processing and presentation and inflammatory response, among others (Table 6).

**Table 6:** Functional annotation of genes regulated in the duodenum of mice fed an iron-supplemented diet.

Functional Category	Gene Symbol	Description	GenBank Number	FC	Q-PCR
Glutatione metabolism	Gstm1	glutathione S-transferase, mu 1	NM_010358	4.42	4.29
	Gsta3	glutathione S-transferase, alpha 3	NM_010356	4.27	
	Gsta1	glutathione S-transferase, alpha 1 (Ya)	NM_008181	3.51	
	Gsta2	glutathione S-transferase, alpha 2 (Yc2)	NM_008182	2.93	
	Gstm6	glutathione S-transferase, mu 6	NM_008184	2.80	
	Gstm4	glutathione S-transferase, mu 4	NM_026764	2.41	
	Gsta4	glutathione S-transferase, alpha 4	NM_010357	2.26	
	Gstm3	glutathione S-transferase, mu 3	NM_010359	1.88	
	Anpep	alanyl (membrane) aminopeptidase	NM_008486	−1.83	
Antigen processing and presentation	Cd74	CD74 antigen (invariant polypeptide of major histocompatibility complex, class II antigen-associated)	BC003476	−1.95	
	H2-Eb1	histocompatibility 2, class II antigen E beta	NM_010382	−2.06	
	H2-DMa	histocompatibility 2, class II, locus DMa	NM_010386	−2.07	
	Psmb8	proteasome (prosome, macropain) subunit, beta type 8 (large multifunctional peptidase 7)	NM_010724	−2.07	
	H2-DMb2	histocompatibility 2, class II, locus Mb2	NM_010388	−2.16	
	H2-Aa	histocompatibility 2, class II antigen A, alpha	NM_010378	−2.53	
	H2-Ab1	histocompatibility 2, class II antigen A, beta 1	NM_207105	−2.76	
Chaperone cofactor-dependent protein folding	Cd74	CD74 antigen (invariant polypeptide of major histocompatibility complex, class II antigen-associated)	BC003476	−1.95	
	H2-DMa	histocompatibility 2, class II, locus DMa	NM_010386	−2.07	
	H2-DMb2	histocompatibility 2, class II, locus Mb2	NM_010388	−2.16	
	Dnajb1	DnaJ (Hsp40) homolog, subfamily B, member 1	NM_018808	−2.62	−2.17
	Hsph1	heat shock 105 kDa/110 kDa protein 1	NM_013559	−5.64	−6.55
MHCII	H2-Eb1	histocompatibility 2, class II antigen E beta	NM_010382	−2.06	
	H2-DMa	histocompatibility 2, class II, locus DMa	NM_010386	−2.07	
	H2-DMb2	histocompatibility 2, class II, locus Mb2	NM_010388	−2.16	
	H2-Aa	histocompatibility 2, class II antigen A, alpha	NM_010378	−2.53	
	H2-Ab1	histocompatibility 2, class II antigen A, beta 1	NM_207105	−2.76	
T cell differentiation and activation	Cd74	CD74 antigen (invariant polypeptide of major histocompatibility complex, class II antigen-associated)	BC003476	−1.95	
	H2-DMa	histocompatibility 2, class II, locus DMa	NM_010386	−2.07	
	H2-Aa	histocompatibility 2, class II antigen A, alpha	NM_010378	−2.53	
	Egr1	early growth response 1	NM_007913	−3.33	−2.32
	Hsp90aa1	heat shock protein 90 kDa alpha (cytosolic), class A member 1	NM_010480	−2.11	
Inflammatory response	Reg3g	regenerating islet-derived 3 gamma	NM_011260	−1.56	
	Cxcl13	chemokine (C-X-C motif) ligand 13	NM_018866	−1.71	
	C3	complement component 3	NM_009778	−1.78	
	Ccl5	chemokine (C-C motif) ligand 5	NM_013653	−2.00	
	Pap	pancreatitis-associated protein	NM_011036	−2.13	
Antimicrobial	Defcr20	defensin related cryptdin 20	NM_183268	1.72	
	Defcr5	defensin related cryptdin 5	NM_007851	−1.41	
	Lyzs	lysozyme	NM_017372	−1.88	
	Defcr-rs1	defensin related sequence cryptdin peptide (paneth cells)	NM_007844	−3.23	
Lectin	Reg2	regenerating islet-derived 2	NM_009043	2.14	
	Glg1	golgi apparatus protein 1	NM_009149	−1.43	
	Reg3g	regenerating islet-derived 3 gamma	NM_011260	−1.56	
	Pap	pancreatitis-associated protein	NM_011036	−2.13	
B cell mediated immunity	C3	complement component 3	NM_009778	−1.78	
	Cd74	CD74 antigen (invariant polypeptide of major histocompatibility complex, class II antigen-associated)	BC003476	−1.95	
	H2-DMa	histocompatibility 2, class II, locus DMa	NM_010386	−2.07	
Cholesterol metabolic process	Ldlr	low density lipoprotein receptor	NM_010700	1.99	
	Cyp51	cytochrome P450, family 51	NM_020010	1.96	
	Hmgcs2	3-hydroxy-3-methylglutaryl-Coenzyme A synthase 2	NM_008256	1.88	
Response to heat	Hspa1a	heat shock protein 1A	NM_010479	−1.91	
	Hsp90aa1	heat shock protein 90 kDa alpha (cytosolic), class A member 1	NM_010480	−2.11	
	Hsph1	heat shock 105 kDa/110 kDa protein 1	NM_013559	−5.64	−6.55

We identified genes whose expression was affected by both *Hfe* deficiency and dietary iron supplementation in the duodenum. There were 4 genes whose expression was induced in both conditions, 3 genes whose expression was decreased, and 4 genes with opposite regulation (Table 7).

**Table 7:** Genes regulated in the duodenum of mice by *Hfe* deficiency or iron-supplemented diet.

	Gene Symbol	Description	GenBank	FC Hfe^−/−^	FC diet
Increased in Hfe^−/−^ and by diet	*Reg2*	regenerating islet-derived 2	NM_009043	10.34	2.14
	*Alpi*	alkaline phosphatase, intestinal	NM_001081082	2.09	1.71
	*Akr1b8*	aldo-keto reductase family 1, member B8	NM_008012	1.60	4.17
	*Mboat1*	membrane bound O-acyltransferase domain containing 1	NM_153546	1.46	1.81
Increased in Hfe^−/−^ and decreased by diet	*Reg3b*	regenerating islet-derived 3 beta	NM_011036	6.87	−2.13
	*Klk1b27*	kallikrein 1-related peptidase b27	NM_020268	2.22	−1.87
	*Slc38a5*	solute carrier family 38, member 5	NM_172479	2.14	−2.31
Decreased in Hfe^−/−^ and increased by diet	*Defcr20*	defensin related cryptdin 20	NM_183268	−2.69	1.72
Decreased in Hfe^−/−^ and by diet	*Hspb1*	heat shock protein 1	NM_013560	−2.07	−2.17
	*Defcr-rs1*	defensin related sequence cryptdin peptide (Paneth cells)	NM_007844	−1.60	−3.23
	*LOC620017*	PREDICTED: similar to Ig kappa chain V-V region L7 precursor	XM_357633	−1.44	−2.31

FC, fold-change; diet, iron-supplemented diet.

#### Altered expression of iron-related genes in the duodenum

**Figure 3:**
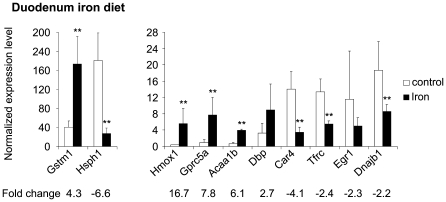
Expression of genes regulated in the duodenum of dietary iron-loaded mice as verified by Q-RT-PCR. Samples from 5 mice fed an iron-supplemented diet and 4 mice fed a control diet were used, and each sample was run in triplicate. (mean±SD). *p<0.05; **p<0.025; ***p<0.01.

In the duodenum of *Hfe^−/−^* mice, *Hamp2* expression was increased by 2.7-fold using microarray analysis. However, this could not be confirmed by Q-RT-PCR, because *Hamp2* mRNA levels in the samples from wild-type mice and in one *Hfe^−/−^* sample were below the detection threshold. In mice fed the iron-supplemented diet, the duodenal expression of *Tfrc* was downregulated and that for *Hmox1* was upregulated: both of these results were validated by Q-RT-PCR (Figure 3).

#### Confirmation of duodenal microarray results by Q-RT-PCR

**Figure 4:**
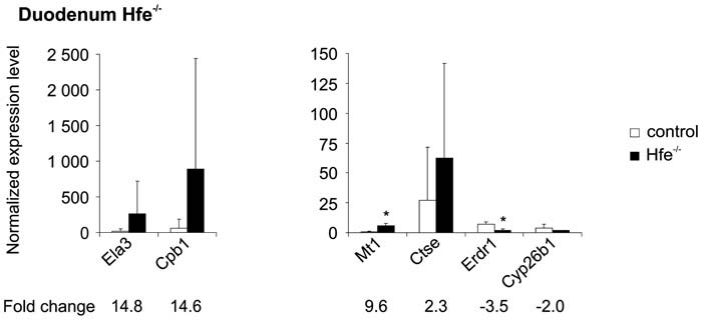
Validation of the duodenal microarray results from *Hfe^−/−^* mice by Q-RT-PCR. The *Hfe^−/−^* and control groups contained samples from 4 mice, and each sample was tested in triplicate. (mean±SD). *p<0.05; **p<0.025; ***p<0.01.

Q-RT-PCR analyses were done on duodenal RNA samples from 5 *Hfe^−/−^* mice, 4 wild-type control mice, 5 iron-fed mice and 4 mice fed a standard diet in order to confirm the microarray results. The mRNA expression of a total of 17 different genes was tested and 16 (94.1%) showed concordant results between microarray analysis and Q-RT-PCR (Figures 3 and 4). The sole discrepant result concerned the expression of *Ddb1* that was downregulated according to microarray analysis, while Q-RT-PCR revealed a slight induction (1.25-fold) of expression.

## Discussion

The goal of this study was to explore and compare the genome-wide transcriptome response to *Hfe* deficiency and dietary iron overload in murine liver and duodenum. This approach allowed the identification of genes whose expression is changed during iron overload and those genes whose expression is differentially influenced by lack of Hfe protein. The global transcriptional response to *Hfe* deficiency has been explored previously in the liver and duodenum of two mouse strains [17]. However, it is notable that only a few analogous changes in gene expression are seen when comparing our data with those of the previous study, even for mice of the same genetic background. Two other reports have explored expression of selected genes by using dedicated arrays in *Hfe^−/−^* mice and in mice with secondary iron overload produced by intraperitoneal injection of iron-dextran [19], [20]. In one study, duodenum and liver samples were analyzed using an array of iron-related genes [19]. The results for duodenal gene expression in *Hfe^−/−^* mice have no concordance with ours. Regulation of hepatic gene expression, on the other hand, is similar for several genes, such as *Hamp1*, *Tfrc* and *Mt1*. The second report focused on gene expression in the duodenum [20], and again, there is little concordance between their observations and ours. The lack of agreement between these studies is probably due to differences in the animal models (parenteral vs. enteral iron loading; mouse strains) and in the microarray methodology.

The hepatic expression of acute phase proteins (APPs) can be induced by inflammatory mediators such as interleukin-6. Interestingly, the liver of *Hfe^−/−^* mice has upregulated expression of APPs such as serum amyloids, lipocalins and orosomucoids. Notably, the expression of serum amyloid genes (*Saa1*, *Saa2*, *Saa3*) was upregulated in the *Hfe^−/−^* mice compared to being downregulated in dietary iron overload, suggesting that *Hfe* deficiency induces this gene expression by an iron-independent mechanism. However, hepatic interleukin-6 mRNA expression was not significantly changed by *Hfe* deficiency, so the potential involvement of this cytokine in the observed upregulation of APPs remains uncertain.

Lipocalin2 (human Ngal from neutrophil gelatinase-associated lipocalin) is an APP with antimicrobial properties through a mechanism of iron deprivation by siderophore binding [21]. It can donate iron to various types of cells [22], [23] and seems to be capable of intracellular iron chelation and iron excretion [24]. Furthermore, a recent study has shown that lipocalin2 is an adipokine with potential importance in insulin resistance associated with obesity [25]. We observed that *Lcn2* expression is increased in the liver of both *Hfe^−/−^* mice and those with dietary iron overload, suggesting that this induction is iron-related.

Dietary iron overload of the liver led to increased expression of both hepcidin genes (*Hamp1*, *Hamp2*) as previously reported [26], [27], and these results were verified by Q-RT-PCR. In the liver of *Hfe* ^−/−^ mice, *Hamp1* expression was downregulated as expected [15], [16], [19]. We also examined the levels of *Hamp2* mRNA by Q-RT-PCR and found a -1.92-fold change. The low expression of hepatic *Hamp1* in *Hfe* ^−/−^ mice is likely responsible for the increased iron absorption and low microphage iron content in these mice [15], [16], [19].

Inhibitor of DNA-binding/differentiation proteins, also known as Id proteins, comprise a family of proteins that heterodimerize with basic-helix-loop-helix (bHLH) transcription factors to inhibit their binding to DNA. Several studies have reported that Id proteins have important roles in differentiation, cell cycle and angiogenesis in various cell types [28]. Expression of *Id1*, *2*, and *3* is increased during liver disease, with levels that escalate as liver disease progresses from hepatitis to cirrhosis. In hepatocellular carcinoma, high expression is observed in well-differentiated tumors, and it decreases as the tumor cells become undifferentiated [29]. In light of these findings, it has been suggested that *Id1*, *2*, and *3* may play a role in the early stages of hepatocarcinogenesis. Given that, it is notable that we found that the expression of *Id1*, *2*, *3*, and *4* was increased in the liver of mice with dietary iron overload, but was unaffected in *Hfe* ^−/−^ mice. Increased hepatic expression of *Id1* mRNA has previously been reported in mice fed an iron-supplemented diet [30]. The same study showed upregulation of the gene for bone morphogenetic protein 6 (*Bmp6*) in the same experimental mice. Recent work demonstrates that Bmp6 is a key player in the signalling pathway that controls hepcidin expression [31]. Unexpectedly, upregulation of hepatic *Bmp6* mRNA expression by dietary iron overload was not evident in the current study.

The gene expression of several heat shock proteins was downregulated in the liver and duodenum by both Hfe deficiency and dietary iron overload, with a considerably greater number of these genes downregulated in the liver of *Hfe−/−* mice. Although these genes are induced under certain stress conditions, such as heat shock and ischemia–reperfusion, their expression is decreased by iron overload [19], [27], [32]. Currently, the physiological implications of this downregulation are unknown.

Our results indicate that disruption of the *Hfe* gene induces the expression of many genes in the duodenum coding for digestive enzymes, such as elastases, carboxypeptidases, trypsins, chymotrypsins, amylases, and lipases. In contrast, feeding mice with an iron-supplemented diet did not affect the expression of any of these genes. The upregulation of gene expression for digestive enzymes in *Hfe* ^−/−^ mice is surprising because overexpression of these enzymes has not been associated with HH.

A common feature of the duodenal response to both *Hfe* deficiency and dietary iron overload was the transcriptional repression of genes involved in antimicrobial activities, such as cryptdins. In mice fed an iron-supplemented diet, there was also a decrease in mRNA expression for genes involved in antigen processing and presentation, such as some genes of the MHC class II family.

The solute carrier molecules constitute a large family of proteins involved in membrane transport of diverse molecules. The gene expression of many family members was affected by *Hfe* deficiency or dietary iron overload. In the duodenum, the expression of the sodium-coupled neutral amino acid transporter *Slc38a5* was induced in *Hfe* ^−/−^ mice and repressed in mice fed an iron-supplemented diet. In the liver, the expression of *Slc46a3* was upregulated in *Hfe* ^−/−^ mice. This gene belongs to the Slc46 subfamily of heme transporters. It is thus a close relative of *Slc46a1* (also known as *HCP1*), a recently identified, although controversial, heme transporter [33], [34]. The iron transporter Dmt1, encoded by *Slc11a2*, contains an iron-responsive element (IRE) in the 3′UTR of its mRNA. This permits the regulation of *Dmt1* mRNA levels according to the cellular labile iron pool by mediation of the iron regulatory proteins, IRP1 and IRP2. Under iron-replete conditions, IRP activity is reduced rendering the *Dmt1* mRNA vulnerable to degradation. The opposite is true under iron-deficient conditions, which is believed to be the situation inside the enterocytes of HH patients and *Hfe* ^−/−^ mice [35], [36]. Accordingly, in some studies, increased expression of *Dmt1* has been observed in the duodenum of HH patients [37] as well as in *Hfe* ^−/−^ mice [38]. However, we did not find a significant change in the expression of *Dmt1* in the *Hfe* ^−/−^ duodenum. This may be explained by the inability of our microarray probes and PCR primers to discriminate between IRE-positive and IRE–negative transcripts.

The post-transcriptional regulation of *Tfrc* (transferrin receptor 1) by iron is also mediated through the IRE/IRP system [39]. Tfrc is involved in the uptake of transferrin-bound iron by cells. Analogous to our observations, suppression of *Tfrc* expression in the duodenum [40] and liver [27] of mice fed an iron-supplemented diet, and in the liver of *Hfe^−/−^* mice [19] has been reported previously. Our microarray analysis indicates that the expression of *Tfrc* was not significantly changed in the duodenum of *Hfe* ^−/−^ mice, a result that agrees with a previous report [19].

Excess free iron increases oxidant production [1]. Subsequently, some antioxidant defense mechanisms are upregulated in order to provide resistance to iron-related toxicity. It is notable from our data that this response is elicited in both liver and duodenum, as seen in the upregulation of glutathione S-transferase genes. Interestingly, dietary iron overload seems to induce a stronger response than *Hfe* deficiency, especially in the regulation of enzymes involved in glutathione-related detoxification of reactive intermediates.

In conclusion, *Hfe* deficiency results in increased gene expression of hepatic APPs and duodenal digestive enzymes. In contrast, dietary iron overload causes a more pronounced change of gene expression responsive to oxidative stress.

## Materials and Methods

### Ethics Statement

The animal protocols were approved by the Animal Care and Use Committees of Saint Louis University and the University of Oulu (permission No 102/05).

### Animal care and animal models

Five male C57BL/6 mice homozygous for a disruption of the *Hfe* gene and 4 male wild-type control mice were fed a standard rodent diet (250 ppm of iron) and sacrificed at approximately 10 weeks of age. The generation of the *Hfe^−/−^* mice has been described elsewhere [13]. In addition, 5 male C57BL/6 mice fed an iron-supplemented diet (2% carbonyl iron) and 4 male control mice fed a standard diet (200 ppm of iron) for 6 weeks were used [27]. The mice with dietary iron overload had a hepatic iron concentration that was approximately 2.5 times higher than the *Hfe^−/−^* mice. The duodenum and liver samples were immediately collected from anesthetized mice and immersed in RNAlater solution (Ambion, Huntingdon, UK).

### RNA isolation

Total RNA extraction and quality control have been described previously [27].

### Microarray analysis

All microarray data reported in the present article are described in accordance with MIAME guidelines, have been deposited in NCBI's Gene Expression Omnibus public repository [41], and are accessible through GEO Series accession number GSE17969 [42]. Microarray experiments were performed in the Finnish DNA Microarray Centre at Turku Centre for Biotechnology using Illumina's Sentrix Mouse-6 Expression Beadchips. Duodenal and liver RNA samples from 3 *Hfe^−/−^* mice and 3 mice with dietary iron overload were used. As controls, RNA samples from the duodenum and liver of 4 wild-type mice (2 controls of the *Hfe^−/−^* mice and 2 controls of the mice with dietary iron overload) were used. All 10 samples were analyzed individually. The amplification of total RNA (300 ng), *in vitro* transcription, hybridization and scanning have been described before [27].

### Data analysis

Array data were normalized with Chipster (v1.1.1) using the quantile normalization method. Quality control of the data included non-metric multidimensional scaling, dendrograms, hierarchical clustering, and 2-way clustering (heat maps). These analyses showed that data from one of the three duodenal samples from *Hfe^−/−^* mice were highly divergent from the other two. Thus, this sample was excluded from further analyses. The data were then filtered according to the SD of the probes. The percentage of data that did not pass through the filter was adjusted to 99.4%, implicating a SD value of almost 3. At this point, statistical analysis was performed using the empirical Bayes t-test for the comparison of 2 groups. Due to the small number of samples, the statistical results were considered as orientative and thus no filtering was applied to the data according to p-values. The remaining 280 probes were further filtered according to fold-change with ±1.4 as cut-off values for up- and down-regulated expression, respectively. The functional annotation tool DAVID (Database for Annotation, Visualization and Integrated Discovery) [43], [44] was used to identify enriched biological categories among the regulated genes as compared to all the genes present in Illumina's Sentrix Mouse-6 Expression Beadchip. The annotation groupings analyzed were: Gene Ontology biological process and molecular functions, SwissProt Protein Information Resources keywords, SwissProt comments, Kyoto Encyclopedia of Genes and Genomes and Biocarta pathways. Results were filtered to remove categories with EASE (expression analysis systematic explorer) scores greater than 0.05. Redundant categories with the same gene members were removed to yield a single representative category.

### Quantitative Reverse-Transcriptase PCR

For this analysis, duodenal and liver RNA samples from 5 mice of each experimental group (*Hfe^−/−^* and dietary iron overload) and 4 mice from each control group (wild-type and normal diet) were used. Exceptionally, for the analysis of mRNA expression in the duodenum of *Hfe^−/−^* mice, only 4 samples were used. RNA samples (5 µg) were converted into first strand cDNA with a First Strand cDNA Synthesis kit (Fermentas, Burlington, Canada) using random hexamer primers. The relative expression levels of target genes in the duodenum and liver were assessed by Q-RT-PCR using the LightCycler detection system (Roche, Rotkreuz, Switzerland). The reaction setup, cycling program, standard curve method and primer pairs for *Angptl4*, *Dnajb1* and *Tfrc* have been described before [27]. Mouse *Hamp1* and *Hamp2* primers have also been characterized previously [26]. The primer sets for the other target genes (Dataset S5) were designed using Primer3 [45], based on the complete cDNA sequences deposited in GenBank. The specificity of the primers was verified using NCBI Basic Local Alignment and Search Tool (BLAST) [46]. To avoid amplification of contaminating genomic DNA, both primers from each set were specific to different exons, when possible. Each cDNA sample was tested in triplicate. The mean and SD of the 3 crossing point (Cp) values were calculated for each sample and a SD cutoff level of 0.2 was set. Accordingly, when the SD of the triplicates of a sample was greater than 0.2, the most outlying replicate was excluded and the analysis was continued with the two remaining replicates. Using the standard curve method, the Cp values were then transformed by the LightCycler software into copy numbers. The expression value for each sample was the mean of the copy numbers for the sample's replicates. This value was normalized by dividing it by the geometric mean of the 4 internal control genes, an accurate normalization method [47]. The normalization factor was always considered as a value of 100 and the final result was expressed as relative mRNA expression level.

### Statistical analyses

We performed statistical analyses of the microarray data using the empirical Bayes t-test for the comparison of 2 groups, and the p-values are shown in supplementary datasets S1-S4. For the Q-RT-PCR results, we used the Mann-Whitney test to evaluate differences in group values for *Hfe^−/−^* mice vs. wild-type mice and mice with dietary iron overload vs. untreated mice. Due to the small sample sizes, the statistical significance is only considered as orientative. Values are expressed as mean±SD.

## Supporting Information

Dataset S1 List of genes differentially expressed in the liver of Hfe knockout mice
(0.04 MB XLS)

Dataset S2 List of genes differentially expressed in the liver of mice fed an iron-supplemented diet
(0.05 MB XLS)

Dataset S3 Genes whose expression was altered in the duodenum of Hfe knockout mice
(0.05 MB XLS)

Dataset S4 Genes whose expression was affected in the duodenum of mice fed an iron-supplemented diet
(0.04 MB XLS)

Dataset S5 Sequences of the primers used in the Q-RT-PCR experiments performed in this study
(0.06 MB DOC)
