# Supplementary material for: Global Transcriptional Response to Hfe Deficiency and Dietary Iron Overload in Mouse Liver and Duodenum
Source: PLoS One. 2009 Sep 29;4(9):e7212. doi: 10.1371/journal.pone.0007212 (PMC2747280; doi:10.1371/journal.pone.0007212)
Supplement: Dataset S5 — Sequences of the primers used in the Q-RT-PCR experiments performed in this study (0.06 MB DOC) [file pone.0007212.s005.doc]

Dataset S1. Sequences of the RT-PCR primers used in this study

| **Symbol** | **Name** | **GenBank Accession No.** | **Forward primer (5'-3')** | **Reverse primer (5'-3')** |
| --- | --- | --- | --- | --- |
| *Acaa1b* | acetyl-Coenzyme A acyltransferase 1B | NM_146230 | TGTCCCAGAGAGGGAACCA | CCTGCTTCTGCCGTGAAAC |
| *Acot3* | acyl-CoA thioesterase 3 | NM_134246 | ACTTTGAGGAAGCTGTGACC | CGCCGATGTTGGATATAGAG |
| *Cp* | ceruloplasmin | NM_001042611 | CAGCCGTAGAGGTGGAATG | TAAACTGGCGATACACAACC |
| *Cpb1* | carboxypeptidase B1 (tissue) | NM_029706 | GGTTTCCACGCAAGAGAG | GTTGACCACAGGCAGAACA |
| *Creld2* | cysteine-rich with EGF-like domains 2 | NM_029720 | GAACGAGACCCACAGCATC | CCACATCCACACAGGCATC |
| *Ctse* | cathepsin E | NM_007799 | CACACCCAGTATTCCATCCA | ATCCACAGTCAACCCTTCCA |
| *Cyp26b1* | cytochrome P450, family 26, subfamily b, polypeptide 1 | NM_175475 | CAAGCTCGGCAGATCCTTCA | ACTCCAGGGTTCCATCCTTC |
| *Cyp2c54* | cytochrome P450, family 2, subfamily c, polypeptide 54 | NM_206537 | TATTGGTGGGACAGAGTCAA | CATTTGTATAGGGCATGTGG |
| *Cyp4a14* | cytochrome P450, family 4, subfamily a, polypeptide 14 | NM_007822 | CAAGACCCTCCAGCATTTCC | CCCAGAACCACCTTCACATAG |
| *Dbp* | D site albumin promoter binding protein | NM_016974 | TGAGGAACAGAAGGATGAGAAG | ACAGCACGGTAGTGGGACAG |
| *Egr1* | early growth response 1 | NM_007913 | AGCGGCGGTAATAGCAGCA | GGGATAACTCGTCTCCACCA |
| *Ela3* | elastase 3, pancreatic | NM_026419 | TGCCTGTGGTGGACTATGAA | CAGCCCAAGGAGGACACAA |
| *Erdr1* | erythroid differentiation regulator 1 | NM_133362 | TTTCTCTGTGGGCGTGAATG | GCAGGCTTCCTACCTTGTG |
| *Gprc5a* | G protein-coupled receptor, family C, group 5, member A | NM_181444 | AGAGCTATGGTGTGGAGAA | CTGAAAATGGGTGGAATAAG |
| *Gstm1* | glutathione S-transferase,  mu 1 | NM_010358 | ACGCCTTCCCAAACCTGA | GGGCCTACTTGTTACTCCA |
| *Hmox1* | heme oxygenase  (decycling) 1 | NM_010442 | CAGAGGAACACAAAGACCAGA | CCAACAGGAAGCTGAGAGTG |
| *Hsd3b5* | hydroxy-delta-5-steroid dehydrogenase, 3 beta- and steroid delta-isomerase 5 | NM_008295 | GTGAGCTGTACCTGCCTTCA | GCACCAACATTCGGACAATC |
| *Hsph1* | heat shock 105kDa/110kDa protein 1 | NM_013559 | TCACCATCTCCACGGCTTC | GCTTCACTGTTGTCTTGCTG |
| *Id2* | inhibitor of DNA binding 2 | NM_010496 | ACCACCCTGAACACGGACA | CTCCTGGTGAAATGGCTGA |
| *Id3* | inhibitor of DNA binding 3 | NM_008321 | ATCTCCAAGGACAAGAGGAG | AGGCGTTGAGTTCAGGGTAA |
| *Lcn2* | lipocalin 2 | NM_008491 | CAATGTCACCTCCATCCTG | CTGGTTGTAGTCCGTGGTG |
| *Ltf* | lactotransferrin | NM_008522 | CGGAGAAGTATCTGGGAAAG | ACAGCAGGGAGTGAGGAGA |
| *Mt1* | metallothionein 1 | NM_013602 | CCTCACTTACTCCGTAGCTC | GCACTTGCAGTTCTTGCAG |
| *Rsad2* | radical S-adenosyl methionine domain containing 2 | NM_021384 | TGGTGCCTGAATCTAACC | TTCTTCCACGCCAACATC |
| *Saa1* | serum amyloid A 1 | NM_009117 | CATTTGTTCACGAGGCTTTC | CGAGCATGGAAGTATTTGTC |
| *Saa2* | serum amyloid A 2 | NM_011314 | TGGTCTTCTGCTCCCTGCTC | GTATTTGTCTCCATCTTTCCAG |
| *Slc46a3* | solute carrier family 46, member 3 | NM_027872 | GTGTGACCAAAACAAAAGCAG | CCCAGAGAGCCAAGAGATG |
